# CT-guided joint cavity release for postpartum sacroiliac joint pain management: an evaluation of its efficacy, safety, and clinical outcomes

**DOI:** 10.3389/fmed.2024.1417673

**Published:** 2024-09-26

**Authors:** Yang Mao-jiang, Qiong Xian, Anup Bhetuwal, Li Bing, Xu Xiao-xue

**Affiliations:** ^1^Department of Pain, Affiliated Hospital of North Sichuan Medical College, Nanchong, Sichuan, China; ^2^The Second Affiliated Hospital of North Sichuan Medical College, Nanchong, Sichuan, China

**Keywords:** sacroiliac joint, postpartum, imaging-guided, release, intervention

## Abstract

**Objective:**

The central aim of this study was to evaluate the safety and effectiveness of Computed Tomography (CT)-guided joint cavity release in treating patients suffering from postpartum sacroiliac joint pain.

**Methods:**

A retrospective analysis was conducted on a sample of 37 patients who presented with postpartum sacroiliac joint pain and underwent CT-guided sacroiliac joint release treatment at The Affiliated Hospital of North Sichuan Medical College. General clinical attributes of the patients were recorded, and the intensity of their pain before and after the operation was compared using the Numeric Pain Rating Scale (NRS). The effectiveness of the surgical treatment was assessed using the Modified MacNab criteria. The functional status of the sacroiliac joint at 3-and 6-month intervals post-operation was examined, and any complications related to surgery were documented.

**Results:**

The follow-up period was completed by all patients, with the successful implementation of CT-guided unilateral/bilateral sacroiliac joint release undertaken in 37 patients. Patient reported pain, as measured by the Numeric Pain Rating Scale (NRS), was considerably reduced postoperatively with scores showing significant decrement from 7.14 ± 1.23 preoperatively to 1.26 ± 0.53 at 1 week, 1.86 ± 0.62 at 1 month, 1.92 ± 0.48 at 3 months, and 1.97 ± 0.61 at 6 months postoperatively, respectively (*p* < 0.05). The comprehensive record of treatment response rates, interpreted as excellent and good, were consistent, standing at 100% (37/37), followed by 97.30% (35/37) and concluding with 91.89% (33/37). The Oswestry Disability Index (ODI) scores reflecting the patient’s perceived level of disability prior to the surgery, and at 3 and 6 month intervals post-surgery were 45.12 ± 6.01, 18.14 ± 2.23, and 14.25 ± 2.15, respectively, demonstrating a significant improvement in postoperative scores when compared with preoperative scores (*p* < 0.05). The surgeries conducted were devoid of any complications such as bleeding, infection, cardiovascular or cerebrovascular incidents, or decline in joint functionality in any of the patients.

**Conclusion:**

Evidently, CT-guided joint cavity release presents as an effective therapeutic approach for the management of postpartum sacroiliac joint pain, enhancing quality of life and preserving patient safety.

## 1 Background

Dysfunction of the sacroiliac joint during pregnancy or in the postpartum period can arise from a multitude of biomechanical changes, such as weight gain, postural adjustments, augmented abdominal and intrauterine pressures, and the loosening of ligaments in spinal and pelvic areas, as documented in the literature (1–3). It is estimated that approximately 50% of women experience sacroiliac joint discomfort during these stages, and although the majority recover within 4 months postpartum, about 20% endure ongoing pain (4, 5). Notably, the incidence of sacroiliac joint pain reported in everyday settings by postpartum women may be underrepresented, as many seek medical intervention only when the pain substantially interferes with their daily activities. This suggests that the actual prevalence of sacroiliac joint pain among postpartum women could be higher than previously estimated, thus necessitating further attention to this condition (6, 7).

Historically, treatment modalities for sacroiliac joint pain have encompassed a range of interventions including physical therapy, pharmacological treatments, nerve blockades, intra-articular injections, radiofrequency ablation, and surgical fixation of the sacroiliac joint, each with variable outcomes (8–10). In the current study, we utilized CT-guided sacroiliac joint release surgery as a treatment for 37 patients experiencing postpartum sacroiliac joint pain, aiming to assess its effectiveness and safety profile. The results are detailed in the subsequent sections.

## 2 Materials and methods

### 2.1 General information

A retrospective study was conducted on a cohort of 37 patients diagnosed with postpartum sacroiliac joint pain, who were treated at the Affiliated Hospital of North Sichuan Medical University over a period extending from February 2018 to May 2022. The study meticulously gathered and statistically scrutinized demographic data and clinical symptoms of these patients (refer to Table 1). The research methodology was rigorously designed to be in strict compliance with the ethical standards outlined in the Declaration of Helsinki, in addition to adhering to the procedural guidelines set forth by the Affiliated Hospital of North Sichuan Medical University. Prior to their inclusion in the study, each patient was thoroughly briefed on the study protocol and treatment procedures, post which informed consent was obtained.

**Table 1:** Demographics characteristics of patients (*n* = 37).

Variables	
Age (year)	32.2 ± 3.4 (21 ~ 44)
Height (cm)	158.4 ± 4.6 (148 ~ 172)
Weight (Kg)	51.4 ± 2.4 (41 ~ 68)
BMI index	
<18.5	2 (5.4%)
18.5–24.9	16 (43.3%)
25–30	17 (45.9%)
>30	2 (5.4%)
Course of disease (month)	13.2 ± 3.4 (24 ~ 56)
Delivery method, n (%)	
Natural childbirth	28 (75.6%)
Cesarean section	9 (24.4%)
Pain frequency, n (%)	
Paroxysmal	32 (86.5%)
Persistence	5 (13.5%)
Pain orientation, n (%)	
Left	14 (37.8%)
Right	16 (43.3%)
Bilateral	7 (18.9%)
Combined symptoms, n (%)	
Lumbar pain	31 (83.8%)
Sacrococcygeal pain	14 (37.8%)
Hip pain	26 (70.2%)
Groin pain	11 (29.7%)
Thigh pain	18 (48.6%)
Calf pain	6 (16.2%)
Restricted activities	22 (59.5%)

The criteria for inclusion in the study were meticulously defined to ensure a homogeneous patient profile. These criteria encompassed: (1) A clinical confirmation of postpartum sacroiliac joint pain, substantiated by symptomatology and corroborated by imaging studies; (2) A history of insufficient symptom relief following conservative treatment approaches, such as oral medication administration and engagement in pelvic floor exercises; (3) A Numeric Rating Scale (NRS) pain intensity score equal to or exceeding 4; (4) Voluntary agreement to undergo the treatment protocol as proposed in the study.

Conversely, potential participants were excluded from the study based on the following criteria: (1) Current breastfeeding status; (2) The presence of lumbar or pelvic masses, as evidenced by MRI or CT scans; (3) The presence of abnormal coagulation function or active anticoagulant therapy, which could predispose to bleeding complications; (4) The existence of an infection at the site designated for surgical intervention. These exclusion criteria were established to mitigate potential confounding factors and ensure patient safety throughout the study.

### 2.2 Methods

#### 2.2.1 Preoperative preparation

**Figure 1:**
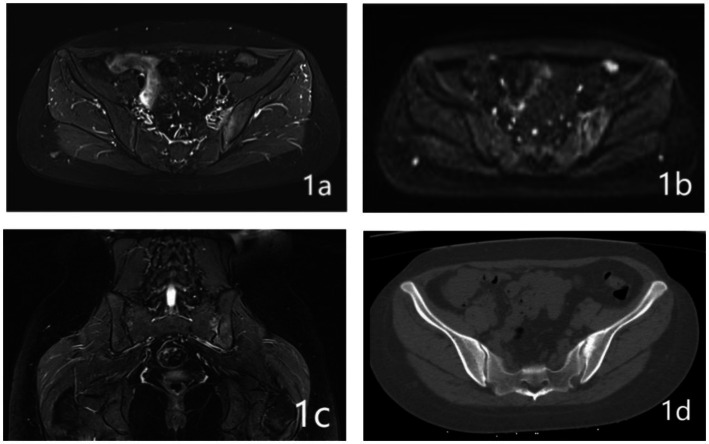
A 32-year-old woman who presented with persistent pain in her left sacroiliac joint, which had been on going for 6 months following childbirth. Pre-operative MRI and CT examination revealed the following: **(A)** The fs-MRI (fat-suppressed MRI) of the left sacroiliac joint displayed an increased signal intensity beneath the joint surface. **(B)** Diffusion-Weighted Imaging (DWI): The DWI scan revealed restricted diffusion in the left iliac bone and the surface of the sacroiliac joint. **(C)** Coronal plane fs-MRI illustrating increased signal beneath the left sacroiliac joint. **(D)** CT cross-sectional analysis: The CT scan of the left iliac bone showed increased bone density.

Upon their admission, patients were subjected to computed tomography (CT) and magnetic resonance imaging (MRI) of the lumbar spine and pelvis. This imaging was essential to accurately identify lesions within the sacroiliac joint, as depicted in Figure 1. Additionally, routine preoperative assessments were diligently performed to exclude any contraindications to the surgical procedure.

#### 2.2.2 Surgical procedures

**Figure 2:**
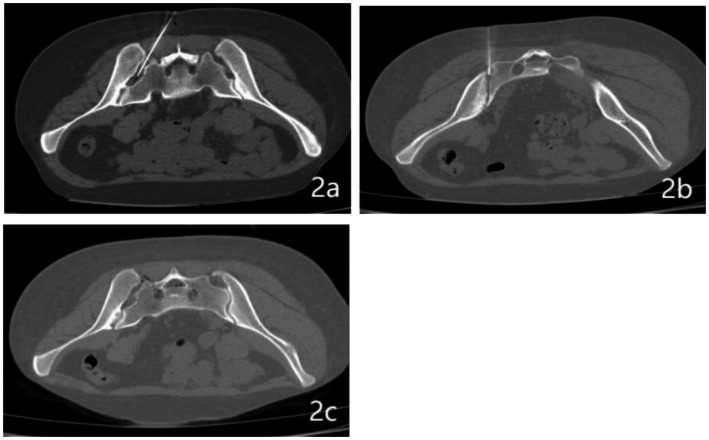
CT-guided joint cavity release surgery. **(A)** Puncture of the left sacroiliac joint cavity utilizing a specialized puncture needle. **(B)** Engagement of the posterior ramus of the sacroiliac joint via puncture needle insertion. **(C)** Detailed visualization of gas distribution both within the sacroiliac joint cavity and its adjacent regions.

Prior to the surgery, patients were required to fast for a duration of 4 h. The procedure was carried out in the MR/CT intervention center of our hospital. Using Philips 64 slice spiral CT with a slice thickness of 1 mm and continuous scanning, each patient is scanned 5–6 times during the operation, with a total radiation dose of approximately 50–60 mGy. During the surgery, patients were placed in a prone position, and their vital signs, including heart rate, blood pressure, pulse rate, and blood oxygen saturation, were continuously monitored. In order to precisely target the sacroiliac joint space and its posterior border, a custom-designed metal fence marker was securely placed on the affected gluteal region. The surgical approach adopted was a posteromedial one, accessed through the gluteal region, and local infiltration anesthesia was administered using 1% Lidocaine. For the purpose of puncturing, a 22G, 14 cm coaxial trocar was utilized. The needle’s advancement was carefully guided by a pre-determined angle and pathway, as illustrated in Figures 2A,B. The correct positioning of the puncture needle was verified through CT scanning. Subsequent to this verification, a combination of 5 mL of ozone and 5 mL of anti-inflammatory fluid (comprising 0.25% Lidocaine, 1 mg of compound Betamethasone, and 1.5 mg of cobalamine adenosine) was injected. A follow-up CT scan was conducted to confirm the effective distribution of ozone within the sacroiliac joint cavity, as shown in Figure 2C. Post-procedure, the puncture needle was removed, and patients were advised to remain in a supine position for 6 h in the ward. To ensure consistency and reduce variability in the surgical procedure, all interventions were performed by the same senior associate chief physician and an attending physician.

### 2.3 Evaluation of treatment efficacy

#### 2.3.1 Pain assessment

The severity of pain both preoperatively and postoperatively was quantitatively assessed using the Numeric Rating Scale (NRS), which spans from 0 (indicating no pain) to 10 (representing the most severe pain). The effectiveness of the treatment was further evaluated through the application of the Modified MacNab Scoring Scale, which classifies outcomes into four distinct categories: excellent, good, fair, or poor. The combined rate of excellent and good outcomes was calculated using the formula: [{Number of Excellent Outcomes + Number of Good Outcomes}/{Total Number of Patients}] × 100%. The criteria for the Modified MacNab Scale are delineated as follows: Excellent: The patient experiences complete alleviation of pain symptoms, facilitating a return to normal work and daily activities. Good: There is a significant reduction in pain levels, allowing for a near-normal resumption of work and everyday life. Fair: The patient experiences a partial reduction in pain, which continues to impact normal work and daily activities. Poor: There is either no improvement in pain levels or an exacerbation of symptoms.

#### 2.3.2 Functional evaluation

The postoperative functionality of the sacroiliac joint was assessed using the Oswestry Disability Index (ODI). This index comprises 10 questions that evaluate various aspects: pain intensity, self-care, social life, lifting, walking, sitting, standing, sleeping, sexual function, and engagement in recreational activities. Each question is assigned a score ranging from 0 to 5, with the cumulative score potentially reaching a maximum of 50 points. A higher total score is indicative of more severe functional impairment. All participants completed this questionnaire postoperatively to facilitate a comprehensive assessment of functional outcomes.

#### 2.3.3 Surgical complications

In our study, we meticulously documented any surgical complications that occurred. These included but were not limited to hemorrhagic events (bleeding), infectious complications, cardiovascular and cerebrovascular incidents, and any observed deterioration in pelvic function.

### 2.4 Statistical methods

For the analysis of our data, we utilized SPSS statistical software, IBM SPSS 25.0 (SPSS Inc., Chicago, IL, United States). Our approach to data presentation involved the use of descriptive statistics. Specifically, categorical data were expressed as percentages, while continuous variables were articulated as means accompanied by standard deviations [denoted as (x \pm s)]. To scrutinize the changes in Numeric Rating Scale (NRS) and Oswestry Disability Index (ODI) scores across various time points within the group, we employed repeated measures analysis of variance. Furthermore, the Least Significant Difference (LSD) test was applied for *post hoc* analysis. A *p*-value threshold of less than 0.05 (*p* < 0.05) was established to determine statistical significance.

## 3 Results

### 3.1 Patient demographics

This study encompassed a cohort of 37 patients. The age distribution among these patients ranged from 21 to 44 years, with the mean age being 32.2 years, accompanied by a standard deviation of ±3.4 years. The duration of symptoms reported by these individuals varied considerably, extending from a minimum of 6 months to a maximum of 4 years, with the average duration calculated at 1.7 years (±0.3 years).

In terms of symptomatology, all patients in the study presented with persistent and recurrent pain, which was localized to the lumbosacral region, gluteal areas, pelvic girdle, or lower limbs. A significant proportion of the cohort, accounting for 31 cases (83.8%), reported experiencing concurrent lumbar and back discomfort. Furthermore, hip pain was a common complaint, noted in 26 cases (70.2%). Additionally, 22 patients (59.5%) exhibited varying degrees of restricted mobility, as detailed in Table 1. To ensure the comprehensiveness of our study, all patients were diligently followed up for a period of 6 months. This follow-up was conducted either through telephonic conversations or during outpatient services. Notably, there were no instances of patients being lost to follow-up during this period.

### 3.2 Evaluation of patient postoperative outcomes

An analysis of preoperative and postoperative Numeric Rating Scale (NRS) scores revealed substantial reductions at intervals of 1 week, 1 month, 3 months, and 6 months following the surgical procedure. These reductions are indicative of a significant alleviation of postoperative pain, as evidenced by the observed statistically significant differences. Moreover, an examination of the Oswestry Disability Index (ODI) scores in the preoperative and postoperative phases disclosed marked improvements at both 3 and 6 months post-surgery when compared to preoperative values. These improvements were statistically significant (*p* < 0.05). However, it is noteworthy that the analysis did not reveal any statistically significant difference in the ODI scores between the 3-month and 6-month postoperative periods (*p* > 0.05), as detailed in Table 2.

**Table 2:** Comparison of NRS and ODI scores during the follow-up period.

Time	NRS	ODI
Preoperative	7.14 ± 1.23	45.12 ± 6.01
1w post-surgery	1.26 ± 0.53*	
1 m post-surgery	1.86 ± 0.62*	
3 m post-surgery	1.92 ± 0.48*	18.14 ± 2.23*
6 m post-surgery	1.97 ± 0.61*	14.25 ± 2.15*#
*F*	6.14	9.51
*P*	0.000	0.000

*Compared with preoperative, *P* < 0.05; #Compared with postoperative 3 months, *p* > 0.05.

### 3.3 Evaluation of clinical efficacy

The assessment of clinical outcomes post-surgery demonstrated that the percentages of “excellent” and “good” results at subsequent intervals of 1 week, 1 month, 3 months, and 6 months were uniformly high, with initial rates being 100% (37 out of 37 patients) at both 1 week and 1 month. These rates slightly decreased to 97.30% (35 out of 37 patients) at the 3-month mark and further to 91.89% (34 out of 37 patients) by 6 months post-operation. Notably, after the 6-month evaluation period, three patients experienced a recurrence of pain, highlighting a decline in the success rate of the surgical outcomes, as detailed in Table 3.

**Table 3:** Excellent and good rates according to modified MacNab clinical evaluation criteria.

Time	Excellent	Good	Medium	Poor	Excellent and good rate (%)
1w post-surgery	35(94.59%)	2(5.41%)	0(0.00%)	0(0.00%)	100%
1 m post-surgery	33(89.19%)	4(10.81%)	0(0.00%)	0(0.00%)	100%
3 m post-surgery	28(75.68%)	7(18.92%)	2(5.41%)	0(0.00%)	97.30%
6 m post-surgery	27(72.97%)	7(18.92%)	3(8.11%)	0(0.00%)	91.89%

### 3.4 Surgical complications

None of the patients experienced bleeding, subcutaneous hematoma, infection, or deterioration of sacroiliac joint function.

## 4 Discussion

The sacroiliac joint (SIJ), forming a union between the sacrum and the bilateral iliac bones, stands as the largest true synovial joint in the human body. Characterized by its uniquely fitting joint surface and an encompassing joint capsule, the SIJ is further stabilized by robust ligaments. These anatomical features collectively confer upon the joint a significant weight-bearing capacity, enabling it to effectively transmit gravitational forces (11). Functionally, the SIJ plays a pivotal role in the biomechanical process of force transference between the spine and the lower limbs. It acts as a critical intermediary in distributing gravitational forces and muscular-generated forces from the surrounding structures to either the lower limbs or the trunk. This distribution is essential for maintaining the overall equilibrium of the body (12). Several key ligaments, including the iliolumbar, sacrospinal, sacral tuberosity, and interosseous sacroiliac ligaments, are integral to the maintenance of the SIJ’s stability. These ligaments not only safeguard the joint’s stability but also permit a degree of micro-motion within the joint, which is crucial for its function (13). In the context of pregnancy, hormonal shifts, particularly the increase in Relaxin and estrogen levels, play a significant role in modulating the SIJ. These hormonal changes induce the relaxation of ligaments surrounding the SIJ, facilitating the expansion of the pelvic band and the stretching of tissues during childbirth. However, these physiological adaptations may lead to potential complications such as separation of the joint surfaces, ligament tears, or even dislocation of the SIJ, culminating in sacroiliac joint pain (14–18).

Contrasting with discogenic lower back pain, patients suffering from postpartum sacroiliac joint pain predominantly exhibit unilateral discomfort inferior to the L5 spinal nerve level. The locus of pain is typically pinpointed at the distal and medial aspects of the posterior superior iliac spine and extends into the medial region of the gluteus. These symptoms, manifesting as tingling, a persistent dull ache, or a burning sensation, are frequently misinterpreted as radicular pain due to their propensity to radiate down to the posterior thigh. This extension notably coincides with the innervation territory of the S1 nerve ganglia, thereby complicating the differential diagnosis (19).

A significant clinical observation in patients with sacroiliac joint pain postpartum is the widespread incidence of pelvic girdle instability. This condition detrimentally affects spinal integrity by compromising the stability of the lumbar region. In our comprehensive study, we examined a cohort of 30 subjects (81.1%) who presented with unilateral symptom onset, 31 individuals (83.8%) who reported lower back discomfort, 18 participants (48.6%) who experienced thigh pain, and 6 patients (16.2%) who described pain extending to the lower leg. Crucially, lumbar magnetic resonance imaging (MRI) assessments, conducted as part of our investigation, revealed lumbar disc herniation and nerve root edema in three cases. These findings highlight the complex and varied nature of postpartum sacroiliac joint pain manifestations.

The initial management of postpartum sacroiliac joint pain predominantly involves conservative methods, including physical therapy, pelvic massage, and pharmacological interventions. However, when these conservative strategies prove insufficient, more invasive treatments may be necessary. Intra-articular and peri-articular corticosteroid injections, as well as radiofrequency ablation, are often employed as secondary interventions (20, 21). Corticosteroid injections aim to alleviate inflammation in the sacroiliac joint and adjacent tissues. Conversely, radiofrequency ablation utilizes thermal energy to disrupt pain transmission by damaging peripheral nerves (22). In cases where these approaches are ineffective, sacroiliac joint fusion surgery becomes a viable option. Although previous research has suggested that sacroiliac joint fusion surgery may be helpful for joint pain, there is a potential risk of postoperative joint stiffness, which may affect daily activities. In addition, it also includes the impact on adjacent joints: stabilizing one joint may increase the pressure on adjacent joints, leading to new problems (23).

In our investigation, we utilized intra-articular and sacroiliac joint posterior branch techniques for ozone nerve release surgery. The underlying mechanism of action encompasses several critical components: Rapid Nerve Conduction Blockade: This is achieved through the application of local anesthetics, facilitating immediate relief from pain. Betamethasone’s Prolonged Effects (24–28): As a glucocorticoid, Betamethasone exerts long-term anti-inflammatory and analgesic impacts. Multifaceted Benefits of Ozone: Ozone therapy offers anti-inflammatory, antioxidant, and analgesic advantages through a variety of mechanisms. These include: (a) Peripheral Nerve Adhesion Release: Ozone acts as a metabolizable gas that promptly alleviates peripheral nerve adhesion. (b) Regulation of Pain Mediators: It modulates the release of nociceptive substances such as 5-hydroxytryptamine, dopamine, and hydrogen dissociation from damaged free nerve endings. (c) Inhibition of Inflammatory Processes: Ozone effectively suppresses the synthesis and activity of protein hydrolases and inflammatory cytokines, facilitating the expression of antioxidant enzymes, neutralizing oxygen free radicals, and thereby safeguarding cellular integrity and fostering the repair of demyelinated nerve fiber bundles. (d) Environmental Optimization within Joint Cavities: By adjusting the pH and osmotic pressure, ozone improves the joint cavity’s internal milieu, thereby promoting cartilage repair. Moreover, ozone therapy has been shown to activate inhibitory interneurons, prompting them to release enkephalin and other pain-relieving substances. Our findings revealed a significant decrease in the Numeric Rating Scale (NRS) and Oswestry Disability Index (ODI) scores during the follow-up period. Notably, we observed excellent and good therapeutic success rates of 97.30 and 91.89%, respectively, at 3 and 6 months following the procedure. These outcomes underscore the substantial therapeutic efficacy of this treatment approach.

Given the intricate nature of accessing deep and confined joint spaces, the role of image-guided interventional therapy has become paramount. CT-guided minimally invasive interventional therapy, in particular, offers considerable benefits in terms of safety, accuracy, efficiency, and effectiveness, establishing it as the preferred modality for pain management (29). In our study, CT-guided sacroiliac joint injections were performed without any incidents of complications, such as bleeding, subcutaneous hematomas, or infections. This success is attributed to thorough preoperative assessments, including magnetic resonance imaging (MRI) and high-resolution computed tomography (CT) scans, which facilitated the early identification of anomalous blood vessels, thereby preemptively mitigating the risk of bleeding. Furthermore, the practice of minimizing repeated needle insertions during the procedure played a crucial role in reducing the likelihood of hematoma formation. Optimal puncture technique, recommending needle insertion parallel to the sacroiliac joint surface to the greatest extent possible, significantly increased the puncture success rate and minimized tissue trauma. This meticulous approach underscores the importance of precision in enhancing treatment outcomes and patient safety in minimally invasive pain management procedures.

### 4.1 Limitations of this study and recommendations for future research

This study has some limitations. Firstly, it includes a relatively small sample of patients. Another reason is that the follow-up time is relatively short. The patient was only followed up for 6 months after injection. In addition, due to the lack of a control group, we cannot rule out bias in patient selection, which may affect the generalizability and reliability of the results. We hope to conduct further research with more patient samples and longer observation periods in the future.

## 5 Conclusion

The conclusion drawn from the study highlights the efficacy and safety of CT-guided sacroiliac joint cavity release surgery as a treatment for postpartum sacroiliac joint pain. This technique has been demonstrated to significantly improve symptoms and enhance the functional status of patients, all while maintaining a high safety profile with no significant complications reported. The use of CT guidance in this context ensures precise targeting and minimally invasive intervention, which are key factors in the successful management of postpartum sacroiliac joint pain. This finding supports the adoption of image-guided surgical interventions as a viable and beneficial option for patients experiencing this specific type of postpartum pain, offering them a path to recovery with minimal risk and maximized therapeutic outcomes.

## Data Availability

The original contributions presented in the study are included in the article/supplementary material, further inquiries can be directed to the corresponding author.
